# Kidney disease among adults on tenofovir-based second-line antiretroviral therapy in Dar es Salaam, Tanzania

**DOI:** 10.4102/sajhivmed.v26i1.1640

**Published:** 2025-01-31

**Authors:** Sabina F. Mugusi, Grace A. Shayo, Philip G. Sasi, Lulu S. Fundikira, Eric A. Aris, Christopher R. Sudfeld, Ferdinand M. Mugusi

**Affiliations:** 1 Department of Clinical Pharmacology, School of Biomedical Sciences, Muhimbili University of Health and Allied Sciences, Dar es Salaam, United Republic of Tanzania; 2 Department of Global Health and Population, Harvard T.H. Chan School of Public Health, Boston, United States of America; 3 Department of Internal Medicine, School of Clinical Medicine, Muhimbili University of Health and Allied Sciences, Dar es Salaam, United Republic of Tanzania; 4 Department of Radiology and Imaging, School of Diagnostic Medicine, Muhimbili University of Health and Allied Sciences, Dar es Salaam, United Republic of Tanzania; 5 Management and Development for Health, Dar es Salaam, United Republic of Tanzania

**Keywords:** HIV, combination antiretroviral therapy, kidney disease, tenofovir, atazanavir sulfate

## Abstract

**Background:**

Kidney disease is a growing non-AIDS-related comorbidity among people living with HIV (PLWH). Tenofovir disoproxil fumarate (TDF) can result in proximal tubulopathy and acute tubular injury, whereas atazanavir/ritonavir (ATV/r) can cause interstitial nephritis and renal stones, both of which can lead to chronic kidney disease.

**Objectives:**

To examine the relationship between second-line combination antiretroviral therapy (ART) and the risk of kidney disease and morphological changes among PLWH in Dar es Salaam, Tanzania.

**Method:**

A cross-sectional study of adult PLWH receiving TDF-based second-line ART. Socio-demographic and clinical data were gathered, and laboratory tests were conducted to determine the estimated glomerular filtration rate (eGFR). Ultrasonography was performed to visualise the kidneys.

**Results:**

A total of 323 patients were enrolled (67.8% women), with a median age of 44 (interquartile range [IQR]: 39–51) years. Patients were on second-line ART for a median of 49 [IQR: 25–73] months, and 60% received ATV/r. Low eGFR (< 90 mL/min per 1.73 m^2^) was found in 22% of patients, proportionately higher among patients on ATV/r compared to those on a lopinavir/ritonavir (LPV/r) (*P* < 0.05). Nearly one-third (32.2%) of patients had a triad of renal calcinosis, renal calculi, and nephritis on ultrasonography. Patients using ATV/r had significantly smaller kidney volumes and greater proportions of renal calculi and nephritis compared to those on LPV/r (*P* < 0.05).

**Conclusion:**

Adults on second-line ART containing TDF were found to have a high prevalence of renal kidney disease in the Tanzanian context. Predictors of kidney disease were older age, proteinuria, and ATV/r-based regimen as compared to LPV/r.

**What this study adds:** This study emphasises the importance of regularly assessing patients on second-line ART for the identified risk factors (age over 45 years, ATV/r-based regimen, and proteinuria) in order to promptly intervene and prevent the development of chronic kidney disease.

## Introduction

The success of combination antiretroviral therapy (ART) and the chronicity of HIV disease has shifted the focus of patient care to chronic conditions, including renal failure.^1^ The prevalence of chronic kidney disease among people living with HIV (PLWH) treated with ART varies greatly, ranging from 2% to more than 30% varying between geographic regions, and based on heterogeneous cut-offs and calculation methods of estimating glomerular filtration rate as a measure of renal function.^2,3,4,5^ Numerous studies have examined the association between several widely used antiretroviral drugs and chronic kidney disease.^6,7,8,9^ However, there is no consensus on the risk of kidney disease associated with HIV infection and the use of ART, but there are data to suggest it may be an important condition that disproportionately affects PLWH.^10,11,12^ Many sub-Saharan African countries, including Tanzania, have introduced tenofovir disoproxil fumarate (TDF) as a preferred nucleotide reverse-transcriptase inhibitor for first- and/or second-line ART. The advantages of TDF include its high potency against HIV and hepatitis B infections, favourable resistance profile, good tolerability and safety, and its availability as a co-formulation with other antiretroviral agents in once-daily pills; however, the risk of kidney disease associated with TDF regimens is not clear.^13,14,15^

Evidence on renal function among individuals on ART regimens containing TDF from randomised controlled trials and observational studies is mixed on whether there is a greater decline in renal function when TDF is co-administered with a boosted protease inhibitor (PI) compared to a non-nucleoside reverse-transcriptase inhibitor (NNRTI) or integrase strand transfer inhibitor (INSTI).^16,17,18,19^ Co-administration of TDF may facilitate the emergence of renal disease related to atazanavir/ritonavir (ATV/r) by contributing to a greater decrease in estimated glomerular filtration rates (eGFR), compared with the use of TDF plus efavirenz (EFV).^20^ Other studies have also shown that exposure to ATV/r was an independent predictor of chronic loss of eGFR among PLWH who had normal renal function at ART initiation, and that this adverse effect was independent of the presence or absence of TDF use.^19^ Calza et al. reported that reduction in eGFR was significantly greater among patients who received ATV/r compared to those who received either EFV or lopinavir/ritonavir (LPV/r), and that the reduction was accompanied by a higher incidence of proximal tubular damage.^21^

Management of comorbidities such as kidney disease can be expensive and may narrow the use of other therapeutic drugs as a result of overt kidney disease. It is therefore important to understand the rates of kidney disease among PLWH in sub-Saharan Africa, along with their risk factors, in order to guide clinical care. The goal of this study was to determine the prevalence of kidney disease among PLWH on second-line ART containing TDF and to assess differences between second-line regimens containing ATV/r and LPV/r in Dar es Salaam, Tanzania.

## Research methods and design

We conducted a cross-sectional study at Muhimbili National Hospital and Amtulabai Kharimjee Clinic HIV clinics in Dar es Salaam, Tanzania. The study enrolled PLWH who were adults (aged > 18 years) and were on a TDF-based second-line ART using combinations of TDF + lamivudine (3TC) + ATV/r or TDF + 3TC + LPV/r. Participants were enrolled between March and June 2017. Written informed consent was obtained from all participants and individuals were excluded if no consent was given.

Socio-demographic and clinical characteristics were recorded in a case report form by the study clinician, which included information on age, sex, marital and education status, smoking and drinking habits, and the use of illicit drugs (substance abuse). Physical examinations included blood pressure and height and weight measurements for body mass index (BMI) calculations. Information on the patient’s past medical history and any comorbidities (such as hypertension and diabetes mellitus) was also collected as well as information from the patients’ medical records to record past drug history/treatments and results of laboratory tests such as serum creatinine, urea, current CD4 cell count, nadir CD4 cell count, and the most recent HIV RNA viral load (within the past 6 months).

Approximately 5 mL of blood was drawn from patients. This sample was transported (in a cool box) and analysed at the Muhimbili University of Health and Allied Sciences Clinical Research Laboratory (MUHAS-CRL). Serum creatinine was quantified using a COBAS INTEGRA^®^ 400 Chemistry Analyzer (Roche Diagnostics International AG, Rotkreuz, Switzerland). A urine sample was also collected for a urine dipstick test (Multistix, Bayer, Leverkusen, Germany) and for microalbuminuria (Clinitek Microalbumin 9, Siemens Healthcare Diagnostics Manufacturing Ltd., Dublin, Ireland). The quantitative analysis of urine albumin levels was done using a Fluorescence Immunoassay (FIA) Rapid Quantitative Test (Finecare™ II FIA Meter, Guangzhou Wondfo Biotech Co., Ltd, Guangzhou, China).

An abdominal ultrasound was conducted using a GE Logiq 9 (General Electric Company, Milan, Italy) ultrasound machine with a trans-abdominal probe of 3.5 MHz – 5 MHz. The ultrasound was done to all the patients by a trained sonologist. The ultrasound scan examined the size and location of both the kidneys, the cortico-medullary differentiation, presence or absence of renal calculi, echotexture, renal pelvis, proximal and distal ureters, and the urinary bladder. The kidney volumes were measured using the ellipsoid formula (length*width*depth*(π/6). In addition, the sonologist also scanned and documented the scans of other organs such as the liver, pancreas, and gall bladder.

### Statistical analysis

We described the proportion of participants with kidney disease based on the eGFR of patients as well as the morphological changes of the kidney. We analysed eGFR based on the Chronic Kidney Disease Epidemiology Collaboration (CKD-EPI) equation for estimating glomerular filtration rate (GFR) expressed for specified race, sex and serum creatinine in μmol/L.^5,22^ For the purposes of our study, we defined our primary outcome as no kidney disease (eGFR ≥ 90 mL/min per 1.73 m^2^) or kidney disease (eGFR < 90 mL/min per 1.73 m^2^). Based on the National Kidney Foundation’s Kidney Disease Outcomes Quality Initiative guideline, CKD is defined as eGFR < 60 mL/min per 1.73 m^2^; however, we wanted to pick up even mildly decreased eGFR (GFR category G2).^5^ For continuous variables, we first tested for normality using the Shapiro-Wilk test. For normally distributed continuous variables, we presented means and standard deviations (s.d.) to describe distributions, and *t*-tests to test differences between groups. Non-normal continuous variables were described as medians with interquartile ranges (IQR) or means with s.d., and the Mann-Whitney U test was used to test differences between groups. Categorical variables were summarised as frequencies and the chi-square test was used to test differences between groups.

We then examined factors associated with kidney disease with eGFR < 90 mL/min per 1.73 m^2^ with logistic regression models. The factors examined included: sex (male, female), age groups (≤ 45 years, > 45 years), BMI (≤ 25 kg/m^2^, > 25 kg/m^2^), duration of ART (≤ 10 years, > 10 years), duration on PI backbone (≤ 24 months, > 24 months), viral load copies (≤ 1000 copies/mL, > 1000 copies/mL), and kidney volumes (> 100 mL, ≤ 100 mL). Other factors included in determining the predictors were the presence or absence of renal stones, nephritis, renal calcinosis, haematuria, and proteinuria. Multivariable models included all the variables used in the univariable analysis. All *P*-values were two-sided values, with < 0.05 considered to be statistically significant. Statistical analyses were performed using Stata version 15 (StataCorp, College Station, Texas, United States).

### Ethical considerations

Ethical clearance was received from the Muhimbili University of Health and Allied Sciences Research and Ethics Committee (reference number 2017-02-22/AEC/Vol.XII/61). Permission to conduct the study at the study site was given by the Municipal Medical Officer in Charge (REF.IMC/DR.6/VolVI/200). Written informed consent was obtained from all patients prior to enrolment into our study. The purpose of the study and the procedures to be done in the study were clearly explained to the patients. Study participants were allocated unique identification codes, and the process of removing personally identifying information was carried out by keeping such information in a distinct and secure database, apart from the other data utilised for analysis. Patients found to have renal dysfunction were reported to the study clinician for a referral to Muhimbili National Hospital Renal Unit for further management.

## Results

A total of 323 adult PLWH on second-line ART were enrolled in the study. The baseline socio-demographic and clinical characteristics of the patients are summarised in Table 1. The median age was 44 years and 67.8% of the patient population were women. The median BMI was 24.1 kg/m^2^ (IQR: 20.9–28.1), and 42.4% were overweight/obese (BMI > 25 kg/m^2^). Patients had been living with HIV for a median of 9 years (IQR: 8–11) and had been receiving second-line ART for a median of 49 months (IQR: 25–73); their median CD4 cell count at the time of switch to second-line ART was 216 cells/mL (IQR: 123–342). The majority (59.4%) of the patients were World Health Organization (WHO) clinical stage III; 17.7% had an unsuppressed viral load > 1000 copies/mL, 5.2% (17/323) had hypertension and 2.2% (7/323) had diabetes mellitus.

**TABLE 1:** Socio-demographic and clinical characteristics of the study patients on tenofovir-based second-line combination antiretroviral therapy (*N* = 323).

Characteristic	*n*	%	Median	IQR¶
**Sex**				
Male	104	32.2	-	-
Female	219	67.8	-	-
**Age groups (years)**	-	-	44	39, 51
≤ 30	14	4.3	-	-
30–45	169	52.3	-	-
> 45	140	43.3	-	-
**BMI (kg/m^2^)**	-	-	24.1	20.9, 28.1
≤ 18.5	27	8.4	-	-
18.5–25.0	155	47.9	-	-
> 25	137	42.4	-	-
**Education**				
No formal education	20	6.2	-	-
Primary	243	75.2	-	-
Secondary	48	14.9	-	-
Post-secondary	12	3.7	-	-
Marital status*				
Single	102	31.6	-	-
Married/cohabiting	152	47.1	-	-
Divorced/separated/widowed	69	21.4	-	-
**Occupation/Employment** ‡				
Unemployed	109	33.8	-	-
Employed	49	15.2	-	-
Self-employed	163	50.5	-	-
**Protease inhibitor ART**				
LPV/r	135	41.8	-	-
ATV/r	188	58.2	-	-
Time on second-line ART (months)	-	-	49	25, 73
Smoking status*				
Current smoker	4	1.2	-	-
Past smoker	50	15.5	-	-
Never smoked	268	82.9	-	-
**Alcohol use**				
Yes	51	15.8	-	-
No	272	84.2	-	-
**WHO clinical stage**				
Stage II	88	27.2	-	-
Stage III	192	59.4	-	-
Stage IV	43	13.3	-	-
Years HIV positive	-	-	9	8, 11
CD4 count at ART initiation	-	-	126	45.5, 235
CD4 count at switch to second-line ART	-	-	216	123, 342
**Current HIV viral load (copies/mL)§**				
≤ 20	66	20.4	-	-
21–1000	120	37.2	-	-
> 1000	57	17.7	-	-

BMI, body mass index; IQR, interquartile range; LPV /r, lopinavir/ritonavir; ATV/r, atazanavir/ritonavir; WHO, World Health Organization; ART, antiretroviral therapy.

* , Missing one observation;

‡ , Missing two observations;

§ , Missing 80 observations;

¶ , quartile 1, quartile 3.

Urine dipstick tests showed that 20% (64/323) of the patients had proteinuria, and 19% (62/323) haematuria. Urinalysis showed that the median urine albumin was 0.58 mg/mmol (IQR: 0.38–1.56). Median serum creatinine was 64.6 μmoles/L (IQR: 54.1–78.3). The calculated median urine albumin creatinine ratio (uACR) was 8.16 mg/mmol (IQR: 5.15–19.71). Using the 2021 CKD-EPI formulae for calculating eGFR, the median eGFR was 108 mL/min per 1.73 m^2^ (IQR: 91–115) (Table 2). The eGFR was significantly higher in patients on the ATV/r regimen compared to those on the LPV/r regimen (*P* = 0.046). The effect size, measured by Cohen’s *d*, was *d* = –0.23 (95% confidence interval [CI]: -0.45-0.01) indicating a small effect. Furthermore, 22% (71/323) of the patients were defined as having kidney disease based on our definitions of eGFR < 90 mL/min per 1.73 m^2^. Patients on the ATV/r regimen were proportionately more likely to have kidney disease than those on LPV/r, with 69% (49/71) of ATV/r patients affected compared to 31% (22/71) of LPV/r patients.

**TABLE 2:** Sonographic and laboratory findings for renal function – overall and stratified by atazanavir/ritonavir (ATV/r) and lopinavir/ritonavir (LPV/r) ART regimen among adults on second-line ART in Dar es Salaam, Tanzania (*N* = 323).

Characteristic	All (*N* = 323)				LPV/r (*n* = 135)				ATV/r (*n* = 188)				*P*
	*n*	%	Median	IQR‡	*n*	%	Median	IQR‡	*n*	%	Median	IQR‡	
**Fatty liver**													0.380
No	69	21.4	-	-	32	23.7	-	-	37	19.7	-	-	
Yes	254	78.6	-	-	103	76.3	-	-	151	80.3	-	-	
**Pancreatic stones**													0.420
No	265	82.0	-	-	108	80.0	-	-	157	83.5	-	-	
Yes	58	18.0	-	-	27	20.0	-	-	31	16.5	-	-	
**Splenic stones**													0.530
No	180	55.7	-	-	78	57.8	-	-	102	54.3	-	-	
Yes	143	44.3	-	-	57	42.2	-	-	86	45.7	-	-	
**Gall bladder stones**													0.840
No	279	86.4	-	-	116	85.9	-	-	163	86.7	-	-	
Yes	44	13.6	-	-	19	43.2	-	-	25	13.3	-	-	
**Renal stones**													0.440
No	104	32.0	-	-	40	29.6	-	-	63	33.7	-	-	
Yes	219	68.0	-	-	95	70.4	-	-	124	66.3	-	-	
**Renal calcinosis**													0.960
No	65	20.1	-	-	27	20.0	-	-	38	20.2	-	-	
Yes	258	79.9	-	-	108	80.0	-	-	150	79.8	-	-	
**Nephritis**													0.520
No	144	44.6	-	-	63	46.7	-	-	81	43.1	-	-	
Yes	179	55.4	-	-	72	53.3	-	-	107	56.9	-	-	
**†Renal volume (mL)**													
Right kidney	-	-	94.5	76.6, 112.9	-	-	97.5	83.9, 117.3	-	-	93	73.2, 111.7	0.018
Left Kidney	-	-	100.9	79.7, 131.1	-	-	110.2	88.4, 136.7	-	-	95	77.2, 128.1	0.005
Laboratory findings													
Urine albumin (mg/mmol)	-	-	0.58	0.38, 1.56	-	-	0.54	0.34, 1.29	-	-	0.63	0.4, 1.91	0.130
Serum creatinine (μmol/L)	-	-	64.6	54.1, 78.3	-	-	64.3	54.1, 77.5	-	-	64.6	53.9, 78.8	0.170
Serum urea (mmol/L)	-	-	18.7	15.3, 23.4	-	-	18.2	15.5, 22.7	-	-	18.8	15.2, 23.5	0.390
eGFR (mL/min per 1.73 m^2^)	-	-	108	91, 115	-	-	107	94, 118	-	-	108.5	87.5, 115	0.046

Note: *P*-value for difference between LPV/r and ATV/r regimens.

LPV/r, lopinavir/ritonavir; ATV/r, atazanavir/ritonavir; ART, antiretroviral therapy; eGFR, estimated glomerular filtration rate; IQR, interquartile range.

† , renal volume calculated using ellipsoid method;

‡ , quartile 1, quartile 3.

Ultrasonographic results are also presented in Table 2 and show that the majority of patients had renal stones (68%), renal calcinosis (80%), and nephritis (55.4%). Renal morphology showed that the left kidney was larger than the right kidney (*P* < 0.05), concurring with normal anatomy where left kidneys are typically longer than the right kidneys. Patients on the ATV/r regimen had significantly smaller kidney volumes for both right (mean ± s.d.; 93 ± 39) and left (95 ± 51) kidneys, compared to those on LPV/r (97.5 ± 33.4 for the right, and 110.2 ± 48 for the left kidney) (*P* < 0.05). Gall bladder stones were also seen in 13.6% of the patients, and splenic stones in 44.3%. Over three-quarters of the patients (78.6%) were found to have a fatty liver. A triad of renal pathology described as having renal stones, together with renal calcinosis and nephritis, were seen in nearly a third (32.2%) of all patients.

The prevalence of kidney disease in our study population was 22% (71/323) as indicated by an eGFR below 90 mL/min per 1.73 m^2^. The majority (50 out of 71) of the patients with kidney disease had mildly decreased eGFR (60–89 mL/min per 1.73 m^2^), whereas 14% (10/71) had mildly to moderately decreased eGFR (45–59 mL/min per 1.73 m^2^), and 11 patients had an eGFR of < 45 mL/min per 1.73 m^2^ and were categorised as having moderate to severely decreased (6/71) and severely decreased (3/71) eGFR. Two patients (2/71) were categorised as having kidney failure resulting from an eGFR of < 15 mL/min per 1.73 m^2^.

**FIGURE 1:**
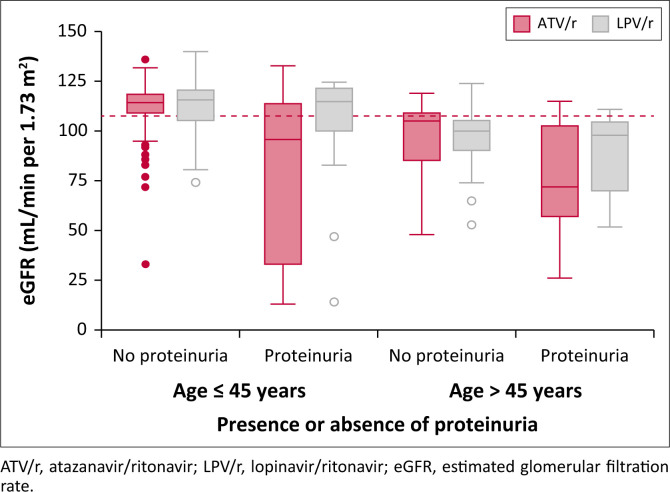
Box plot of estimated glomerular filtration rate clustered according to the protease inhibitor backbone (ATV/r or LPV/r), presence or absence of proteinuria and age group (≤ 45 years or > 45 years of age). Dashed line represents the median eGFR.

In the univariate analysis, age over 45 years, being on an ATV/r-based PI and having proteinuria, renal calcinosis, haematuria, a high uACR, and a smaller left kidney were all found to be associated with kidney disease (eGFR < 90 mL/min per 1.73 m^2^). No association was found with sex, BMI, duration on ART, and duration of PI-based ART, HIV viral load copies, or the presence of sonographic pathologies such as renal stones, nephritis, and renal calcinosis. Multivariable logistic regression analysis identified older age (over 45; adjusted odds ratio [AOR] = 3.63; 95% CI: 1.59–8.25; *P* = 0.002), being on a ATV/r regimen (AOR = 2.37; 95% CI: 1.08–3.67; *P* = 0.03), and having proteinuria (AOR = 2.95; 95% CI: 1.05–8.29; *P* =0.04) as risk factors for kidney disease (Table 3). While not statistically significant, patients with renal calcinosis demonstrated a tendency to have lower eGFR levels (*P* = 0.06). Figure 1 shows the box plots of eGFR clustered according to the PI backbone (ATV/r or LPV/r), presence or absence of proteinuria, and age group (≤ 45 years or > 45 years of age).

**TABLE 3:** Association of demographic, HIV and ART factors with the risk of kidney disease (eGFR < 90 mL/min per 1.73 m^2^) among adults on second-line ART in Dar es Salaam, Tanzania (*N* = 323).

Characteristic	eGFR < 90 Yes (*N* = 71)		Univariate			Multivariate		
	*n*	%	Odds ratio	95% CI	*P*	Odds ratio	95% CI	*P*
**Sex**								
Female	51	23.3	Ref	-	-	Ref	-	-
Male	20	19.2	0.78	0.44–1.40	0.410	0.57	0.24–1.35	0.210
**Age (years)**								
≤ 45	29	15.9	Ref	-	-	Ref	-	-
> 45	42	30.0	2.28	1.33–3.89	0.003	3.63	1.59–8.25	0.002
**BMI (kg/m^2^)**								
≤ 25	43	23.5	Ref	-	-	Ref	-	-
> 25	28	20.4	0.81	0.47–1.39	0.450	0.51	0.22–1.15	0.110
**Duration on ART (years)**								
≤ 10	30	20.3	Ref	-	-	Ref	-	-
> 10	41	23.4	1.21	0.70–2.07	0.680	1.53	0.70–3.32	0.290
**Duration on PI (months)**								
≤ 24	16	20.8	Ref	-	-	Ref	-	-
> 24	55	22.4	1.09	0.58–2.09	0.280	1.63	0.61–4.38	0.330
**PI backbone**								
LPV/r	22	16.3	Ref	-	-	Ref	-	-
ATV/r	49	26.1	1.81	1.03–3.17	0.040	2.37	1.08–3.67	0.030
**HIV viral load (copies/mL)**								
≤ 1000	37	19.9	Ref	-	-	Ref	-	-
> 1000	16	28.1	1.57	0.79–3.10	0.190	1.83	0.76–4.38	0.180
**Renal stones**								
No	25	24.0	Ref	-	-	Ref	-	-
Yes	46	21.0	0.84	0.48–1.46	0.540	0.93	0.42–2.12	0.880
**Nephritis**								
No	34	23.6	Ref	-	-	Ref	-	-
Yes	37	20.7	0.84	0.49–1.43	0.530	0.82	0.38–1.79	0.620
**Renal calcinosis**								
No	8	12.3	Ref	-	-	Ref	-	-
Yes	63	24.4	2.30	1.04–5.08	0.040	3.19	0.94–10.8	0.060
**Proteinuria**								
No	45	17.4	Ref	-	-	Ref	-	-
Yes	26	40.6	3.25	1.79–5.89	< 0.001	2.95	1.05–8.29	0.040
**Haematuria**								
No	50	19.2	Ref	-	-	Ref	-	-
Yes	21	33.9	2.16	1.17–3.98	0.010	1.36	0.49–3.79	0.560
**Urine albumin-creatinine ratio (mg/mmol)**								
≤ 3	40	18.5	Ref	-	-	Ref	-	-
> 3	31	28.9	1.96	1.12–3.45	0.020	0.92	0.37–2.28	0.860
**Volume right kidney (mL)**								
> 100	22	17.5	Ref	-	-	Ref	-	-
≤ 100	49	25.0	1.58	0.89–2.76	0.110	1.59	0.68–3.69	0.280
**Volume left kidney (mL)**								
> 100	26	15.4	Ref	-	-	Ref	-	-
≤ 100	45	29.8	2.28	1.32–3.94	0.003	1.51	0.69–3.32	0.300
**Comorbidities**								
No	66	22.1	Ref	-	-	Ref	-	-
Yes	5	20.8	0.93	0.33–2.58	0.890	0.38	0.07–1.98	0.250

BMI, Body mass index; LPV/r, lopinavir/ritonavir; ATV/r, atazanavir/ritonavir; ART, antiretroviral therapy; PI, protease inhibitor; eGFR, estimated glomerular filtration rate; CI, confidence interval.

## Discussion

In this cross-sectional study, we aimed to determine the prevalence of kidney disease among PLWH on TDF-based second-line ART, and to describe kidney morphology. Defining kidney disease as eGFR < 90 mL/min per 1.73 m^2^, we found a prevalence of 22% in our patient population. Predictors of kidney disease were found to be older age (> 45 years), being on an ATV/r-based PI backbone, and having proteinuria. Overall, nearly a third of our patient population were found to have a triad of renal pathology described as having renal stones, together with renal calcinosis and nephritis.

Renal involvement and the prevalence of kidney disease among PLWH is highly variable, and these conditions are associated with a higher risk of developing end-stage renal disease as compared to HIV-uninfected populations.^23^ Our study found a prevalence of kidney disease at 22%, which is lower than a study by Msango et al. in Mwanza, Tanzania, that found a 38% prevalence.^24^ This difference in prevalence may also be due to the use of different eGFR estimation methods and definitions of kidney disease. However, our findings were similar to the prevalence of 15.8% that was reported by Valdivia-Cerda et al. from a Mexican cohort.^25^ The prevalence of kidney disease appears to vary across different populations but may also be attributed to the different methods of assessments of eGFR as well as the cut-off ranges of the eGFR. A cut-off of < 90 mL/min per 1.73 m^2^ was selected, as we wanted to include mild forms of kidney disease presenting in our patient population. Previous research suggests that among HIV patients using TDF an eGFR of < 90 mL/min per 1.73 m^2^ at the time of discontinuation of TDF was not completely reversible.^26,27^ Therefore, regular estimation of GFR among patients using TDF could allow earlier intervention.

We found that use of TDF co-administered with ATV/r had a higher risk of development of kidney disease as compared to LPV/r. Aligned with our findings, Rasch et al. found that patients in a Danish cohort with baseline eGFR < 90 mL/min per 1.73 m^2^ exposed to TDF and ATV/r in combination had a higher risk of incident CKD compared to patients using TDF and indinavir.^28^ Likewise, Young et al. demonstrated that the use of TDF plus ATV/r contributes to a greater decrease in eGFR, as compared with the use of TDF plus EFV.^20^ Further, in this study, amongst PLWH with a normal baseline renal function (eGFR > 90 mL/min per 1.73 m^2^), rates of decreasing eGFR were high with TDF, ATV/r and LPV/r, and this risk increased with each year of exposure to these ARVs.^8^ Taken in combination with our findings, there is generally consistent evidence that TDF in combination with ATV/r increases the risk of adverse renal consequences.

It has been previously reported that PIs may cause nephrotoxicity and kidney disease.^29,30,31^ ATV/r tends to crystallise in tubular cells, leading to nephrolithiasis and interstitial nephritis, and it is thought that this intratubular crystal formation plays a role in the decrease in eGFR.^32,33^ Both LPV/r and ATV/r increase plasma levels of TDF by 20% – 30% through greater absorption of the prodrug TDF by PI-related inhibition of P-glycoprotein.^31,34,35^ Some researchers suggest that ritonavir inhibits the active tubular secretion of TDF rather than a direct kidney effect.^30,36,37^ Our study has shown that nearly two-thirds of our study population had renal stones, with 40% presenting with stones in the gall bladder, spleen, or pancreas. Observational studies reveal an increased incidence of lithiasis in patients treated with ATV compared to other antiretroviral regimens; however, there was no difference in lithiasis between patients using ATV/r compared to LPV/r.^29,38^ Patients presenting with lithiasis should be monitored more closely, as this is an indication of tubulointerstitial damage resulting from formation of crystals, which could contribute to kidney disease in the long run.

Proteinuria was also found to be a risk factor for kidney disease in our study population. Calculating and reporting of eGFR (generally without consideration of proteinuria) is used to identify patients at risk of kidney disease. However, a study by Garg et al. found that 25% of patient population with proteinuria have overtly reduced eGFR and a similar proportion of those with low eGFR had proteinuria.^39^ Hemmelgarn et al. demonstrated that a patient with a relatively higher eGFR (e.g. 80 mL/min per 1.73 m^2^) and proteinuria (3+ on dipstick) would be assigned to stage 1 CKD, even though their age-adjusted risk would be 2–10 times higher than another patient with a lower eGFR (such as 50 mL/min per 1.73 m^2^), with no evidence of proteinuria classified as stage 3.^40^ Therefore, this suggests that low eGFR and proteinuria do not always coexist, suggesting that eGFR and proteinuria could be used together to identify individuals at high risk for kidney disease.^40^ This also suggests that using an additional dipstick urinalysis may add prognostic information.^41^ Several studies have shown an increased frequency of proteinuria among patients receiving TDF-containing ART, and this proteinuria may precede GFR loss and may be an early sign of kidney disease among patients with normal eGFR.^42,43^

Patients with an older age (> 45 years of age) in our study had more than three times higher risk of having kidney disease. This is consistent with other studies and literature which report that older age was the most important risk factor for the occurrence of kidney disease.^44,45,46,47^ Literature has also shown that kidney function and structure would deteriorate with age.^48,49^ As a result, as PLWH populations are ageing due to expanded ART access, HIV treatment programmes in Tanzania and similar settings should acknowledge that there is a growing need to assess kidney function.

The study had several important limitations. First, this was an observational study and there thus remains a risk of unmeasured or residual confounding. For example, opportunistic infections and their related treatments (such as treatments that could be nephrotoxic) and renal profile prior to initiating ART and before being switched to the second-line ART may be confounders of the relationship of the second-line regimen with renal function. Second, many of the patients in the analysis had been using ART for years and may have been exposed to multiple ART regimens during this period; as a result, the renal damage should not be considered to be fully attributable to the second-line regimens. Third, multiple equations can be used to calculate the eGFR, which may result in some differences between studies. Lastly, we only used one measure of eGFR and proteinuria, and there may therefore be some error in values that may be reduced in future studies by providing multiple measurements.

## Conclusion

In conclusion, kidney disease is common among PLWH on second-line ART containing TDF in Dar es Salaam, Tanzania. We found that older patients and those on ATV/r had a notably higher risk of kidney disease. Proteinuria was also identified as a marker of kidney disease and studies should be done to evaluate if proteinuria could be used as a low-cost way to identify patients at risk for kidney disease. Due to the high prevalence of kidney disease, regular assessment for the risk factors mentioned among patients on second-line ART is important. This will allow for timely interventions and prevention of chronic kidney disease, thereby improving patient care.
